# Drought, Low Nitrogen Stress, and Ultraviolet-B Radiation Effects on Growth, Development, and Physiology of Sweetpotato Cultivars during Early Season

**DOI:** 10.3390/genes13010156

**Published:** 2022-01-16

**Authors:** Purushothaman Ramamoorthy, Raju Bheemanahalli, Stephen L. Meyers, Mark W. Shankle, Kambham Raja Reddy

**Affiliations:** 1 Geosystems Research Institute, Mississippi State University, Mississippi State, MS 39762, USA; purush@gri.msstate.edu; 2 Department of Plant and Soil Sciences, Mississippi State University, 117 Dorman Hall, P.O. Box 9555, Mississippi State, MS 39762, USA; rajubr@pss.msstate.edu; 3 Department of Horticulture and Landscape Architecture, Purdue University, West Lafayette, IN 47907, USA; slmeyers@purdue.edu; 4 Pontotoc Ridge-Flatwoods Branch Experiment Station, North Mississippi Research and Extension Center, Mississippi State University, Pontotoc, MS 38863, USA; mark.shankle@msstate.edu

**Keywords:** aboveground parameters, gas exchange, nutrient deficiency, roots, solar radiation, transpiration efficiency, water stress

## Abstract

Drought, ultraviolet-B (UV-B), and nitrogen stress are significant constraints for sweetpotato productivity. Their impact on plant growth and development can be acute, resulting in low productivity. Identifying phenotypes that govern stress tolerance in sweetpotatoes is highly desirable to develop elite cultivars with better yield. Ten sweetpotato cultivars were grown under nonstress (100% replacement of evapotranspiration (ET)), drought-stress (50% replacement of ET), UV-B (10 kJ), and low-nitrogen (20% LN) conditions. Various shoot and root morphological, physiological, and gas-exchange traits were measured at the early stage of the crop growth to assess its performance and association with the storage root number. All three stress factors caused significant changes in the physiological and root- and shoot-related traits. Drought stress reduced most shoot developmental traits (29%) to maintain root growth. UV-B stress increased the accumulation of plant pigments and decreased the photosynthetic rate. Low-nitrogen treatment decreased shoot growth (11%) and increased the root traits (18%). The highly stable and productive cultivars under all four treatments were identified using multitrait stability index analysis and weighted average of absolute scores (WAASB) analyses. Further, based on the total stress response indices, ‘Evangeline’, ‘O’Henry’, and ‘Beauregard B-14’ were identified as vigorous under drought; ‘Evangeline’, ‘Orleans’, and ‘Covington’ under UV-B; and ‘Bonita’, ‘Orleans’, and ‘Beauregard B-14’ cultivars showed greater tolerance to low nitrogen. The cultivars ‘Vardaman’ and ‘NC05-198’ recorded a low tolerance index across stress treatments. This information could help determine which plant phenotypes are desirable under stress treatment for better productivity. The cultivars identified as tolerant, sensitive, and well-adapted within and across stress treatments can be used as source materials for abiotic stress tolerance breeding programs.

## 1. Introduction

Sweetpotato (*Ipomoea batatas* [L.] Lam.) is the third most crucial storage root crop worldwide, following potato and cassava, with a total production of 91.8 million metric tons (MMT) from 7.8 million hectares (Mha) and an average yield per acre of 9.85 MT. Among the significant sweetpotato-producing countries, the United States is ranked 9th, with a production of 1.45 MMT [1]. Sweetpotato storage roots are considered an essential human diet due to their nutritional quality and fibers (of which 40% is soluble fiber, which helps to lower sugar and cholesterol in the blood), which make it the ideal food for people with diabetes, pregnant women, and children [2,3]. Moreover, it is recognized as a cheap source of energy and vital nutrients to many in developing countries [4,5]. Sweetpotatoes originated in Central and South America and are cultivated worldwide, primarily throughout tropical and subtropical Asia and Africa, in hot semiarid regions where the possibilities of abiotic stresses are acute. In addition, climate change limits crop production by increasing the intensity of abiotic stresses, such as drought, ultraviolet-B (UV-B) radiation, and inadequate soil nutrients [6,7,8].

Sweetpotato production is an essential agribusiness [1] cultivated in rainfed and irrigated environments [9]. Crops grown under rainfed conditions have a higher risk of undergoing drought stress (DS) if the seasonal rainfall fails, which is often combined with stress factors such as low-nitrogen stress (LN) or elevated UV-B radiation. DS negatively impacts plant growth and development based on the intensity and time of occurrence during crop growth. The early stage of plant growth is susceptible to DS, which causes significant morphological, physiology, and molecular changes, resulting in a substantial reduction in plant vigor or economic losses. Sweetpotato is grown primarily in sandy soils and is moderately tolerant of DS [10,11,12]. However, it is still sensitive to DS at the early establishment phase, affecting early vine development and storage root initiation [13]. Earlier studies reported that DS limits the plant water uptake and affects nitrogen availability or uptake. For example, DS reduces nitrogen uptake due to reduced transpiration [14,15]. Though the mineral nitrogen is present in the soil, the absorption of nitrogen by roots is significantly affected by the limited soil moisture content. This interferes with the transportation of solutes from the soil to the roots and shoots. At early seedling stages, DS-induced reduction in the uptake or transport of nitrogen to aboveground tissues simultaneously affects leaf expansion and leaf photosynthesis [16], resulting in poor plant vigor, often leading to crop failure. 

Apart from the abiotic stresses that impact at the soil level, the effects of climate change increase the UV-B radiation (280–315 nm) reaching the Earth’s surface due to the depletion of the stratospheric ozone layer [17,18]. This has led to a significant concern about the impact of enhanced UV-B radiation on plant life, particularly crops [19,20,21]. UV-B radiation alters plant physiological and metabolic processes by affecting the photosynthetic apparatus, resulting in the decreased efficiency of photosystem II by accelerating the chlorophyll degradation [22,23]. Though plants develop a wide range of defensive strategies such as DNA repair; the synthesis of UV-B absorbing compounds such as flavonoids, anthocyanin, and carotenoids; and the thickening of leaves to compensate for the damage caused by UV-B radiation [24,25,26], the ability to sustain these defensive mechanism differs within and across crop species [27]. Numerous studies have demonstrated that UV-B stress inhibits photosynthesis capability [28,29,30]. However, maximum yield potential relies on the early plant vigor to support optimum photosynthesis, which helps initiate and enlarge storage roots in sweetpotato crops. Any disturbance in the photosynthetic rate and other closely associated traits such as stomatal conductance would eventually affect the yield-related features.

In the field, the occurrence of single-stress conditions is rare. Combined stress factors at the early vegetative stage affect several morphological, physiological, and biochemical processes in the plants, such as plant vigor, plant growth rate, assimilate partitioning, photosynthesis, stomatal conductance, translocation, leaf nitrogen, sink strength, and nutrient metabolism [15,31,32,33,34,35]. Recent studies reported that early-season abiotic stress determines root initiation, and mid- and late-season stresses determine biomass and yield in sweetpotatoes [13,36]. However, sweetpotato cultivars respond differently to stress. Understanding cultivar or trait responses to different abiotic stresses is vital for improving plant adaptability under a rapidly changing climate [37]. Compared to direct yield-based evaluation, trait-based evaluation is the best approach to identify stable cultivars, as yield is highly prone to genotype–environment interaction. As a storage root crop, sweetpotato needs to produce more root systems to capture resources to support early plant establishment and develop more sinks as a form of storage roots. The conversion rate of adventitious roots to storage roots at the early stage of crop growth is essential and one of the potential traits in attaining a higher yield. However, the conversion rate can be affected by abiotic stress, and plants often produce pencil roots rather than storage roots. So far, very few studies have reported the effect of DS and UV-B at early crop establishment on storage root initiation and development with only two to three cultivars [33,35]. 

To our awareness, no studies have evaluated the impact of abiotic stress (DS, LN, and UV-B) on root morphology, storage root initiation, and other relevant traits influencing the final root yield using diverse sweetpotato genotypes. Identifying trait responsiveness to each of these stresses could help improve yield-related traits under abiotic stress conditions. Therefore, the current study aims to: (i) assess cultivar responses to DS, LN, and UV-B stress, and (ii) identify traits associated with storage root development and identify stable cultivar(s) across stresses.

## 2. Materials and Methods

### 2.1. Plant Material and Crop Husbandry

The study was conducted in the soil-plant-atmosphere research (SPAR) units at the Rodney Foil Plant Science Research facility of Mississippi State University, Mississippi State, MS, USA (33.28° N, 88.46° W). The growth chambers consisted of a steel soil bin (2m long by 0.5 m wide by 1 m deep) and a Plexiglas chamber (2 m long by 1.5 m wide by 2.5 m tall) to accommodate the pots and plant canopy, respectively. The chambers allowed 97% of the visible solar radiation to pass without spectral variability in absorption. The growth chambers had the capacity to precisely control the irrigation and nutrient supplies, UV-B radiation, atmospheric CO_2_ concentration, and air temperature [38]. The UV-B radiation treatment was possible in the chambers because the SPAR Plexiglass is opaque to solar UV-B.

Field-grown step tip cuttings (slips) of 10 sweetpotato cultivars (Beauregard B-63, Orleans, NC05-198, Bonita, Beauregard B-14, Vardaman, O’Henry, Evangeline, Travis, and Covington), each containing four nodes, were transplanted into polyvinyl chloride (PVC) pots (4″ diameter and 18″ height) on 27 June 2017. The slips were transplanted so that each pot contained a single slip with two nodes (with two recently fully expanded leaves) above and two nodes below the soil surface. A total of 160 PVC pots were arranged in 10 rows, four pots per row in a completely randomized design with 3 × 10 factorial arrangements, where each cultivar was replicated four times within the treatment. Pots were filled with topsoil/sand (1:3, *v*/*v*) medium classified as a sandy loam (87% sand, 2% clay, and 11% silt), with 500 g of gravel at the bottom.

### 2.2. Treatments

The study consisted of a control treatment and three stress treatments: (1) a control, irrigated with full-strength Hoagland’s solution, throughout the experiment (optimal N, 100% irrigation, and natural solar radiation); (2) N reduced in the nutrient solution to 80% of the control (20% N); (3) irrigation reduced to 50% of the control (50% DS) as DS treatment; and (4) 10 kJ UV-B m^−2^ d^−1^. The 20% N treatment was imposed one day after transplanting (DAP) by modifying the standard nutrient solution, substituting CaCl_2_ for Ca(NO_3_) to allow a lower N concentration, as described by Reddy et al. [39]. The 50% DS treatment was imposed 7 DAP by adjusting the time and thus the amount of irrigation based on evapotranspiration (ET), as described by Gajanayake et al. [36]. The 10-kJ m^−2^ d^−1^ of UV-B radiation was delivered at the top of the plant canopy from about 0.5 m above the plant canopy for 8 h, each day, from 08:00 to 16:00 h, by eight UV-B 313 lamps (Q-Panel Company, Cleveland, OH, USA) mounted horizontally on a metal frame inside each SPAR chamber, driven by 40 W dimming ballasts, as described by Reddy et al. [40]. Air temperature (30/22 °C; day/night), CO_2_ concentration (420 ppm), and soil moisture content (100% ET for control or 50% ET for DS treatment) in each chamber were monitored and adjusted every 10 s throughout the day and night with a dedicated computer system, as Reddy et al. [38] described.

### 2.3. Photosynthesis, Fluorescence, and Leaf Pigment Parameters

At 20 DAP, photosynthetic rate (Pn) and fluorescence (Fv’/Fm’) in light were measured on the uppermost second recently fully expanded leaf of each plant between 10:00 and 12:00 h on sunny days using an LI-COR 6400 portable photosynthesis system integrated with a fluorescence chamber head (LI-COR 6400-40 leaf chamber fluorometer, Li-COR Inc., Lincoln, NE, USA). During all the measurements, the instrument was set at a photosynthetically active radiation (PAR) of 1500 µmol (photon) m^–2^ s^–1^, and temperature in the leaf cuvette was set to a daytime temperature of 30°C, with 400 ppm CO_2_ and 50% relative humidity. Pn and Fv’/Fm’ were recorded as the total coefficient of variation (CV, %) reached a value of less than 0.5. By considering the incoming and outgoing flow rates and leaf area, the traits, viz., stomatal conductance (gs), transpiration (E), and electron transport rate (ETR), were calculated by the instrument. The ratio of internal (Ci) to external (Ca) CO_2_ concentration was estimated as the ratio of Pn/E and Ci/Ca. Fv’/Fm’ was calculated and determined as (Fm’ − Fs)/Fm’ by the software in the instrument [33,41].

At 20 DAP, the uppermost recently expanded leaves were used to measure the chlorophyll, flavonoid index, anthocyanin index, and nitrogen balance index (chlorophyll/flavonoid ratio) nondestructively, using a portable, in situ apparatus, Dualex (FORCE-A, Orsay, France).

### 2.4. Growth and Developmental Measurements

The main vine length (VL) and leaf number (LN) were determined at the harvest 20 DAP. The total leaf area (LA) was determined using the LI-3100 leaf area meter (Li-COR) and expressed as cm^2^ per plant. Plants were kept in a forced-air oven at 75 °C until a constant dry weight was reached to determine leaf weight, stem weight, and root weight. Storage root numbers were counted before acquiring the root images. Storage roots were identified as the thickened regions of the primary root that are more than 3 mm in diameter.

### 2.5. Root Sample Extraction and Processing

Roots were washed thoroughly by putting on a wire-mesh sieve and using a slow-speed water stream. The longest root length was measured using a centimeter ruler. Each root system was floated on a waterproof Plexiglas tray and scanned using a specialized dual-scan optical scanner (Regent Instruments Inc., Montreal, QC, Canada). The image acquisition parameters were set to “high” resolution (800 × 800 dpi), and the acquired images were analyzed using WinRHIZO Pro software (Version 2009c; Regent Instruments, Montreal, QC, Canada) to obtain cumulative root length, root surface area, root diameter, root volume, number of tips (root tips), number of forks (root forks), and number of crossings.

### 2.6. Stress-Tolerance Indices

The stress response indices were calculated according to the procedure described in earlier studies [42,43]. First, individual stress response indices (ISRI) were calculated for the three stresses, as the value of a parameter at DS (Pd), LN (Pln), and UV-B (Puv) for a given cultivar divided by the value for the same parameter (Pc) at control condition (Equations (1)–(3)). Then, total DS (Equation (4)), LN (Equation (5)), and UV-B (Equation (6)) stress response indices (TDRI, TLNRI, and TUVRI) were calculated for each cultivar as the sum of all 28 ISRIs in each treatment.

IDRI = P*d*/P*c* (1)

ILNRI = P*ln*/P*c* (2)

IUVRI = P*uv*/P*c* (3)

(4) $$\mathrm{TDRI}=\left(\frac{Pd1}{Pc1}\right)+\left(\frac{Pd2}{Pc2}\right)+\left(\frac{Pd3}{Pc3}\right)\ldots \ldots \ldots +\left(\frac{Pd28}{Pc28}\right)$$

(5) $$\mathrm{TLNRI}=\left(\frac{Pln1}{Pc1}\right)+\left(\frac{Pln2}{Pc2}\right)+\left(\frac{Pln3}{Pc3}\right)\ldots \ldots \ldots +\left(\frac{Pln28}{Pc28}\right)$$

(6) $$\mathrm{TUVRI}=\left(\frac{Puv1}{Pc1}\right)+\left(\frac{Puv2}{Pc2}\right)+\left(\frac{Puv3}{Pc3}\right)\ldots \ldots \ldots +\left(\frac{Puv28}{Pc28}\right)$$

### 2.7. Statistical Analysis

The replication-wise data on the root, shoot, physiological, and gas-exchange traits measured at 20 DAP under DS, LN, and UV-B were subjected to statistical analysis separately, along with the common control treatment using ANOVA. The significance of means was estimated through the F value for each trait. The means derived from ANOVA were used for correlation analysis via GENSTAT (12th ed.) and box plots using SIGMA plot software. Multitrait stability index analysis was carried out using singular value decomposition of the matrix of BLUPs for the genotype–treatment interaction effects generated by a linear mixed model to quantify the stability of each genotype. Each cultivar’s stability was quantified by estimating the weighted average of absolute scores (WAASB) from the singular value decomposition of the matrix of best linear unbiased predictions for the genotype–treatment interaction effects generated by a linear mixed-effect model. Simultaneous selection for mean performance and stability was performed by using the WAASB index, weighting between mean performance (Y) and stability (WAASB) [44]. The genotype with the lowest multitrait stability index (MTSI) value is closer to the ideotype and therefore presents a high mean performance and stability across treatments for all traits studied. The desirable cultivars with maximum productivity and increased stability were selected with 20% selection intensity. These selected and nonselected cultivars were shown graphically by plotting MTSI scores. The studied cultivars were grouped into four classifications through a storage root number–WAASB biplot, which allowed the joint interpretation of stability and mean performance across different stress treatments. This biplot with four quadrants was constructed with storage root number on the x-axis and WAASB scores on the y-axis. The cultivars that possessed a high WAASB score compared to the WAASB grand mean were considered as the most unstable ones. MTSI was carried out in the R studio program (Version 4.11, Integrated Development for R. RStudio, Boston, MA, USA) by using ‘GGEBiplotGUI’ [45] and ‘metan’ [46] R packages.

## 3. Results and Discussion

### 3.1. Photosynthesis and Fluorescence

**Figure 1:**
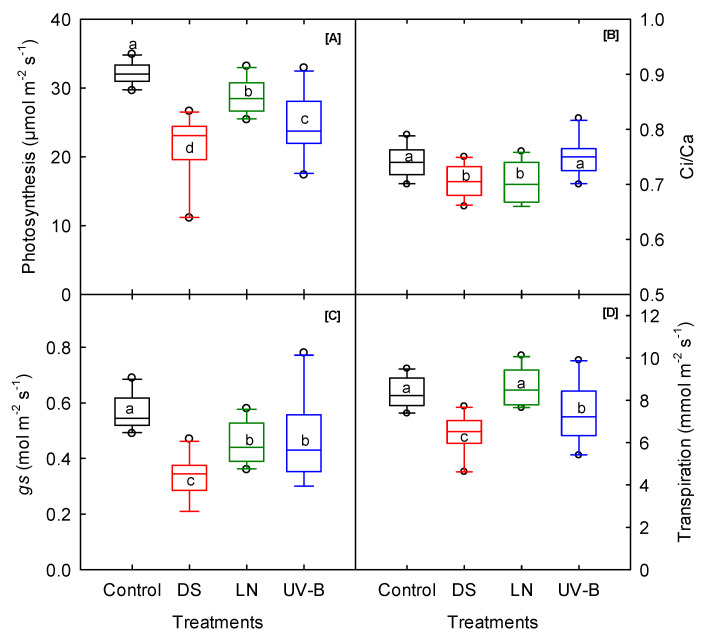
Drought (DS), low nitrogen (LN), and ultraviolet-B (UV-B) effect on (**A**) photosynthesis, (**B**) internal to atmospheric CO_2_ concentration ratio (Ci/Ca), (**C**) stomatal conductance (gs), and (**D**) transpiration of 10 sweetpotato cultivars measured at 20 days after planting. The middle line indicates the median, and the box shows the range of the 25th to 75th percentiles of the total data. The different alphabetic letters (a, b, c, and d) labeled within the box represent the significant differences between treatments (Duncan Multiple Range Test, *p* < 0.05), and the outer dots are outliers.

The treatments significantly differed for photosynthesis, stomatal conductance, ETR, and maximal fluorescence (Table 1). The photosynthesis and stomatal conductance parameters of the sweetpotato cultivars decreased in responses to all three stresses (Figure 1). Compared to the control, DS induced a more significant reduction in the gas-exchange traits, ranging from 21% (ETR) to 42% (stomatal conductance) compared to the control condition. Stomata are essential portals for gas and water exchange in plants and strongly influence characteristics associated with photosynthesis and transpiration [47,48,49,50,51]. A decline in Ci/Ca was only observed under DS and LN compared to control (Figure 1B). In this study, the highest reduction in stomatal conductance (42%) was associated with the maximum decline in transpiration rate (23%) under DS, thus reducing the photosynthetic rate (Figure 1A,C,D). ‘O’Henry’ under DS, ‘NC05-198’ under UV-B, and ‘Orleans’ under LN had the highest photosynthetic rate among the cultivars. Among the cultivars, ‘Vardaman’, ‘Evangeline’, and ‘Orleans’ had the highest stomatal conductance and transpiration rate, and ‘O’Henry’, ‘Beauregard B-14’, and ‘Orleans’ had the highest ETR under DS, UV-B, and LN, respectively (Table S1). However, there were some variations among the cultivars for the traits of minimal fluorescence (Fo), maximal fluorescence (Fm), steady-state fluorescence (Fs), and quantum efficiency (Fv/Fm). ANOVA revealed no significant treatment for the cultivar and cultivar–treatment interaction across treatments, indicating no considerable changes in trait expression between the stress and control among the cultivars (Table 1). Under LN stress, a high increase in Fo, Fm, and Fs, but not in Fv/Fm, was observed. The rise in Fo might have occurred due to irreversible damage in PSII [52], and a similar response was also found in sweetpotatoes grown under heat stress [53].

### 3.2. Leaf Pigments

**Figure 2:**
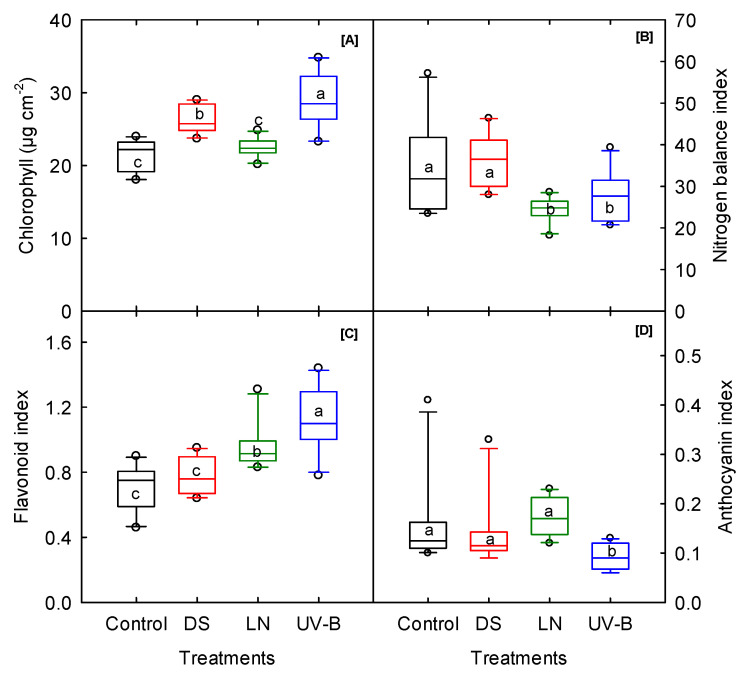
Drought (DS), low nitrogen (LN), and ultraviolet-B (UV-B) effect on (**A**) chlorophyll concentration, (**B**) nitrogen balance index, (**C**) flavonoid index, and (**D**) anthocyanin index of 10 sweet potato cultivars measured at 20 days after planting. The middle line indicates the median, and the box shows the range of the 25th to 75th percentiles of the total data. The different alphabetic letters (a, b, and c) labeled within the box represent the significant differences between treatments (Duncan Multiple Range Test, *p* < 0.05), and the outer dots are outliers.

Averaged over the cultivars, DS and UV-B significantly increased chlorophyll (23% and 35%) and flavonoids (11% and 60%), and at the same time, UV-B reduced anthocyanin by 42% compared to the control (Figure 2A–D). A similar pattern was found under DS, with a 23% increase in chlorophyll and a 13% decrease in anthocyanin (Figure 1). The nitrogen balance index (NBI) indicates the ratio of both the chlorophyll and flavonoids of leaves. This parameter reflects the nitrogen status and health of the plant [54]. The NBI recorded a significant cultivar–treatment interaction (Table 1). Averaged over the cultivars, the NBI significantly reduced under LN (29%) and UV-B (20%) compared to control (Figure 2B). The impact of UV-B on chlorophyll was found to be different, according to a few earlier studies (on rice and maize), where UV-B radiation decreased the pigments [55,56,57,58,59]. It was also reported that some plant species remain unaffected by UV-B by developing protective mechanisms, such as enhancing the antioxidant system [60] and accumulating UV-absorbing compounds [61,62]. Therefore, understanding the pigment modification is necessary to develop UV-B tolerance in plants through a molecular and conventional approach. Among the cultivars, ‘O’Henry’ under DS and UV-B and ‘Travis’ under LN had the highest chlorophyll content. ‘Vardaman’ exhibited significantly less chlorophyll and flavonoid content across the stresses (Table S1).

### 3.3. Shoot Morphological Traits

**Figure 3:**
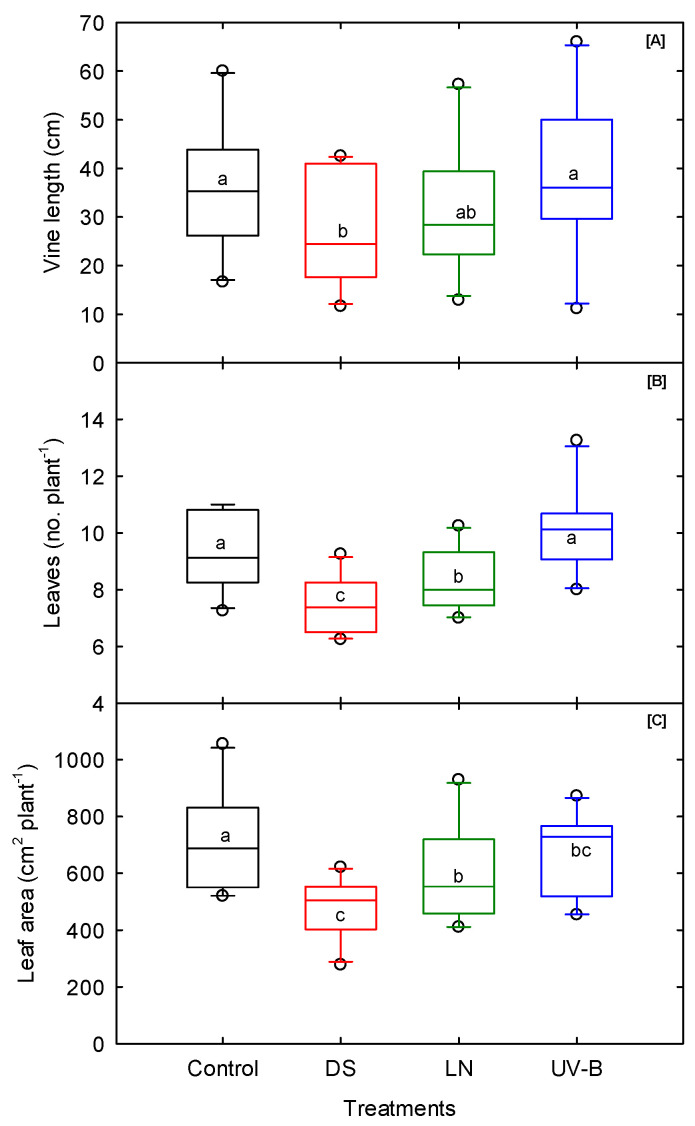
Drought (DS), low nitrogen (LN), and ultraviolet-B (UV-B) effect on (**A**) vine length, (**B**) leaf number, and (**C**) leaf area of 10 sweet potato cultivars measured at 20 days after planting. The middle line indicates the median, and the box shows the range of the 25th to 75th percentiles of the total data. The different alphabetic letters (a, b, and c) labeled within the box represent the significant differences between treatments (Duncan Multiple Range Test, *p* < 0.05), and the outer dots are outliers.

DS, UV-B, and LN caused markable cultivar differences in all the shoot traits (except leaf number) and showed significant cultivar–treatment interactions, except in leaf area, dry leaf weight, stem dry weight, and total dry weight under LN stress (Table 1). Averaged over the cultivars, DS significantly reduced the vine length (24%), leaf number (20%), leaf area (33%), and shoot dry weight (33%) of all the cultivars, followed by LN stress, with a moderate reduction rate ranging from 2% (leaf dry weight) to 17% (stem dry weight) compared to control treatment (Figure 3; Table S1). The leaf area and shoot dry weight components displayed significant cultivar differences (*p* < 0.001) across treatments (Table 1).

Reduced vine length and leaf number in response to water deficit affected the total leaf area and, hence, the total dry weight of the plants [63]. Compared to DS and LN stress, UV-B increased the vine length and leaf number (8%). Plants exposed to DS have a reduced cell division and cell expansion rate, leading to decreased internal nodal elongation and plant height. When the root begins to sense the soil water-deficit stress, it sends a chemical signal into the shoot to minimize excessive water loss [64,65,66]. The overall observations implied that different cultivars exhibited differential DS, UV-B, and common nitrogen stress responses. Thus, their performance would differ according to the variations in water and nutrient availability. ‘Evangeline’ produced the longest vine and highest leaf number under DS and UV-B treatments. In contrast, under LN stress, ‘Beauregard B-14’, ‘Beauregard B-63’, ‘NC05-198’, and ‘Vardaman’ produced the shortest vine lengths and lowest leaf numbers across treatments (Table S2). The cultivars ‘Bonita’ under DS and LN and ‘Orleans’ under UV-B stress exhibited the highest shoot dry weight. The cultivars ‘Covington’ under DS, ‘Beauregard B-14’ under UV-B, and ‘Evangeline’ under LN stress were found to produce the lowest shoot dry weights.

### 3.4. Root Morphological Traits

**Figure 4:**
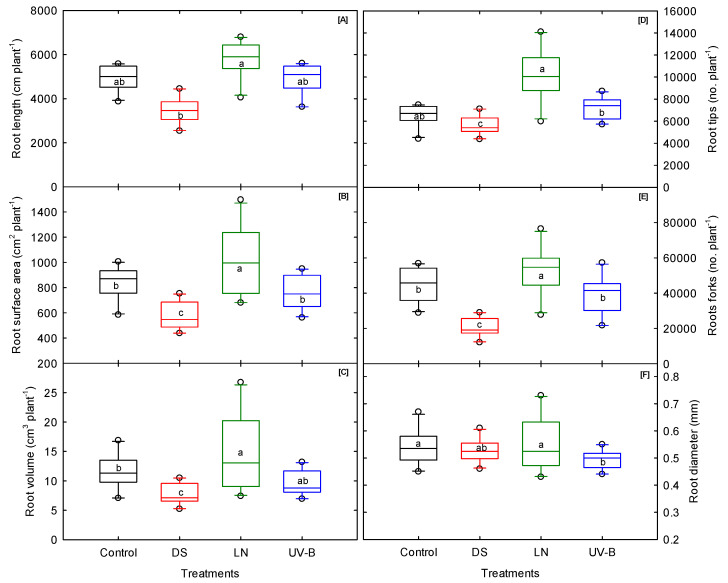
Drought (DS), low nitrogen (LN), and ultraviolet-B (UV-B) effect on (**A**) root length, (**B**) root surface area, (**C**) root volume, (**D**) root tips, (**E**) root forks, and (**F**) root diameter of 10 sweet potato cultivars measured at 20 days after planting. The middle line indicates the median, and the box shows the range of the 25th to 75th percentiles of the total data. The different alphabetic letters (a, b, and c) labeled within the box represent the significant differences between treatments (Duncan Multiple Range Test, *p* < 0.05), and the outer dots are outliers.

In this study, most of the root morphological traits showed no significant cultivar–treatment interaction, but they did show considerable variation among the cultivars in each treatment (Table 1). The cultivars exposed to DS showed a significant decrease in total root length, root surface area, root volume, and root forks (Figure 4A–C,E). The average root diameter was significantly lower under UV-B than control and LN treatments (Figure 4F). Unlike the shoot traits, LN stress increased all the root morphological traits, ranging from a 2% increase (average root diameter) to a 56% increase (root tips). A reduction in the soil nitrogen triggers the foraging response. As a result, the roots grow as they seek nitrogen in the soil profile [67,68]. UV-B stress decreased the root volume (17%) and had minimal effect on the rest of the root traits. The root conversion efficiency is dependent on the sink strength or the size and capacity of the source (leaves) to export the photosynthetic byproducts to the root system. In sweetpotatoes, photosynthates translocated to the root system are partly used to expand fibrous, nonstorage roots, and the rest are deposited in the storage root [69]. The adventitious roots begin to grow within 24 hours of transplanting [33]. Depending on the environmental conditions, storage, pencil, and fibrous roots are produced from the adventitious roots [70].

The expression of root developmental traits varies or is determined by the type of stress the cultivars are exposed to. Stress influences the morphological changes in plant roots, and the rate of these changes depends on the stress intensity [63]. For example, the ‘Covington’ and ‘Evangeline’ cultivars produced high total root length, root volume, and root surface area under UV-B (Table S2). However, the same cultivars had poor root-vigor traits such as total length, root volume, root surface area, and other root parameters under DS and LN stress. Under LN, it was evident that the increase in root surface area was an adaptive trait in low-nitrogen soil [71]. Apart from the storage root number, traits such as root length, surface area, and average root diameter can be considered a proxy to screen sweetpotato cultivars for early seedling vigor. Considering the above three proxy traits, ‘Orleans’ and ‘O’Henry’ under DS; ‘Evangeline’ and ‘Covington’ under UV-B; and ‘Bonita’ and ‘Orleans’ under LN stress were identified as superior trait donors. On the other hand, ‘Vardaman’ was consistently inferior across the three stresses. When comparing the mean values of the root parameters among stresses, there was clear evidence that DS suppressed most of the root-growth-related traits, followed by UV-B and LN stress. Additionally, the same pattern was found in the storage root number, which indicates that root vigor at the early stage of plant growth is necessary to achieve yield improvement under DS.

### 3.5. Biomass and Storage Root Number

**Figure 5:**
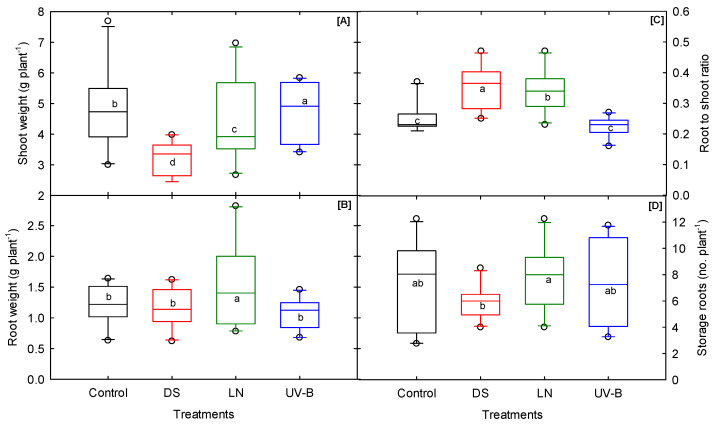
Drought (DS), low nitrogen (LN), and ultraviolet-B (UV-B) effect on (**A**) shoot weight, (**B**) root weight, (**C**) root to shoot ratio, and (**D**) storage root number of 10 sweet potato cultivars measured at 20 days after planting. The middle line indicates the median, and the box shows the range of the 25th to 75th percentiles of the total data. The different alphabetic letters (a, b, c, and d) labeled within the box represent the significant differences between treatments (Duncan Multiple Range Test, *p* < 0.05), and the outer dots are outliers.

A significant cultivar–treatment (DS, LN, and UV-B) interaction was observed for shoot dry weight (Table 1). DS affects growth and development traits depending on the duration and intensity of the stress [72,73,74,75,76]. Compared to the other two treatments, DS had a more significant impact on shoot weight (Figure 5A). The vegetative growth of the sweetpotato cultivars significantly increased under LN and UV-B (Figure 5A). A similar response was found in broad beans and wheat, with the plants’ dry mass increasing or remaining unchanged with rising UV-B [77]. However, a few studies have also reported a significant reduction in leaf area and total dry weight in the sweetpotato cultivar ‘Beauregard’ [35]. These findings suggest that the effect of UV-B is cultivar-specific and that it sometimes benefits the growth and development of a plant canopy [78]. Interestingly, the root dry weight remained unchanged under control and DS conditions and moderately decreased under UV-B (11%), whereas a significant increase in root weight (29%) was observed under LN (Figure 5B). Plants grown under DS and LN had a higher root to shoot ratio, indicating a significant translocation of assimilates from shoot to root (Figure 5C). This implies that the amount of assimilates allocated in the roots was higher under DS and LN stress. The storage root number varied among cultivars across treatments (Table 1). A significant cultivar–treatment interaction (DS and LN) was recorded (Table 1). ‘Covington’ exhibited low to moderate shoot growth and biomass accumulation; it produced the most significant number of storage roots compared to other cultivars under DS and LN (Table S2). This indicates that root growth was favored at the expense of shoot growth or biomass due to decreased soil moisture conditions. A similar response was found in a previous study, wherein the sweetpotato root growth was less affected than the shoot growth under DS [33]. Under LN stress, ‘Orleans’ remained the highest producer of storage root numbers. ‘Travis’ under control, ‘Beauregard B-14’ under DS, ‘O’Henry’ under UV-B, and ‘Vardaman’ under LN stress produced the lowest number of storage roots. DS caused a significant reduction in storage root numbers up to 18% (Figure 5D). However, UV-B stress and LN reduced storage root number production compared to the control treatment. This indicates DS can cause some detrimental impact on early storage root growth and development compared to other abiotic stresses. In general, the cultivars which produced the highest storage root numbers tended to have moderate to low shoot-developmental traits, which was showcased in the cultivar ‘Covington’ (Table S2).

### 3.6. Multitrait Stability Analysis of Sweetpotato Cultivars

**Figure 6:**
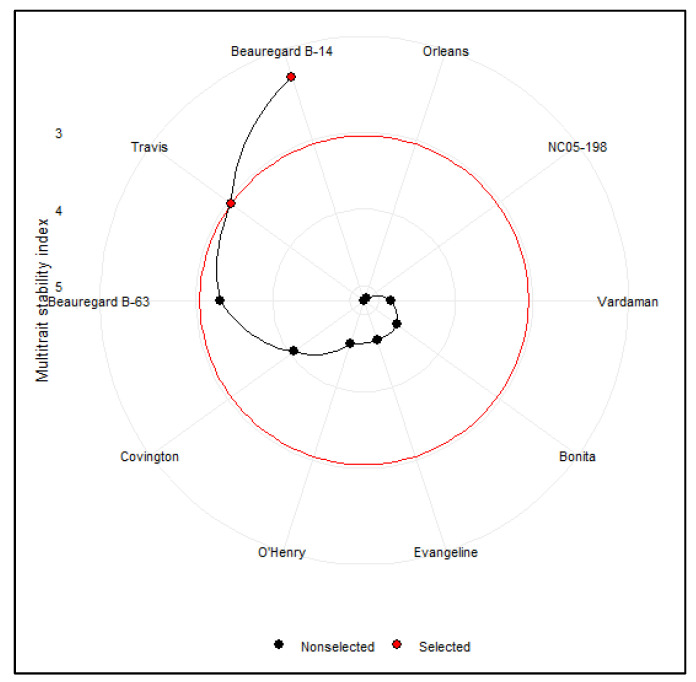
Genotype raking of cultivars based on the multitrait stability index of ten sweetpotato cultivars evaluated under drought, low-nitrogen, ultraviolet-B, and control treatments. The selected genotypes based on this index are shown in red, and the central red circle represents the cutpoint according to the selection intensity (20%).

**Figure 7:**
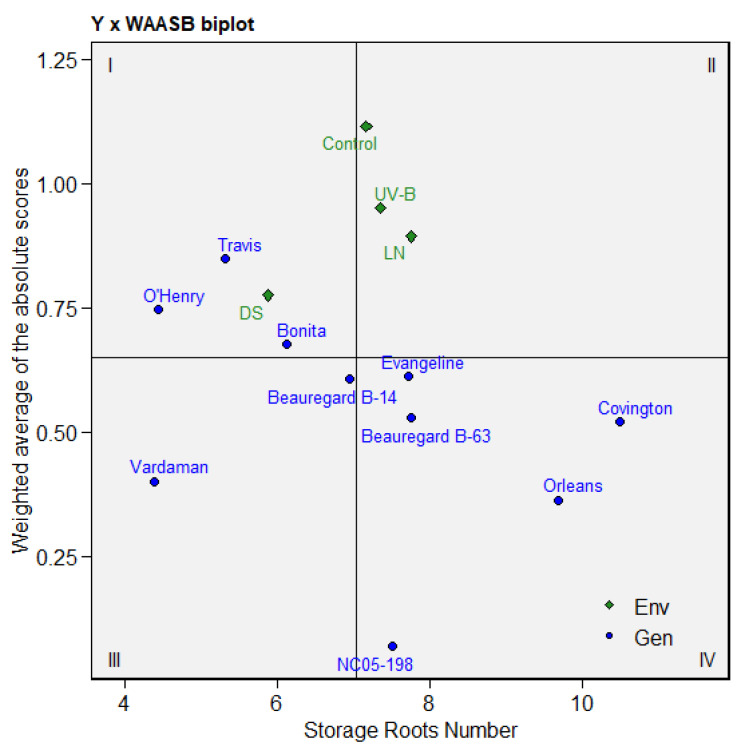
The yield × weighted average of absolute scores (WAASB) biplot based on joint interpretation of storage root number (Y) and stability (WAASB) for ten sweetpotato cultivars evaluated under drought (DS), ultraviolet-B (UV-B), low-nitrogen (LN), and control treatments.

The cultivars that performed well in one or more than one treatment could have combination traits. Based on the multitrait stability analysis, at a selection intensity of 20%, the cultivars ‘Beauregard B-14’ (MTSI = 2.14) and ‘Travis’ (MTSI = 3.05) emerged as the most stable cultivars across treatments (Figure 6). The cultivars with a high MTSI index showed poor performance across treatments and low stability. Given the selection intensity, the ‘Travis’ cultivar was placed on the cut point (Figure 6, red circle). Thus, the cultivars next to the cut point could be considered in the future cultivar improvement program. The quadrant in Figure 7 represents the performance/stress treatments of the four classes of sweetpotato cultivars for the joint interpretation of the storage root numbers and stability of the cultivars. The first quadrant shows that three cultivars (Travis, O’Henry, and Bonita) contributed significantly to the cultivar–treatment interactions. Still, only one treatment (DS) displayed a higher discrimination ability for storage root number (Figure 7).

Any cultivars in the second quadrant are expected to be highly productive but unstable. No cultivars were found in this quadrant, but it did contain three treatments, viz., the control, UV-B, and LN. The two cultivars (Vardaman and Beauregard B-14) that appeared in the third quadrant are considered to be low productive and more stable because of the low WAASB for the storage root numbers across treatments. The cultivars ‘Evangeline’, ‘Beauregard B-63’, ‘NC05-198’, ‘Orleans’, and ‘Covington’ were found in the fourth quadrant with an above-average number of storage roots and lower values of WAASB that indicate broad adaptability.

### 3.7. Cumulative Stress Response Index of Sweetpotato Cultivars

**Figure 8:**
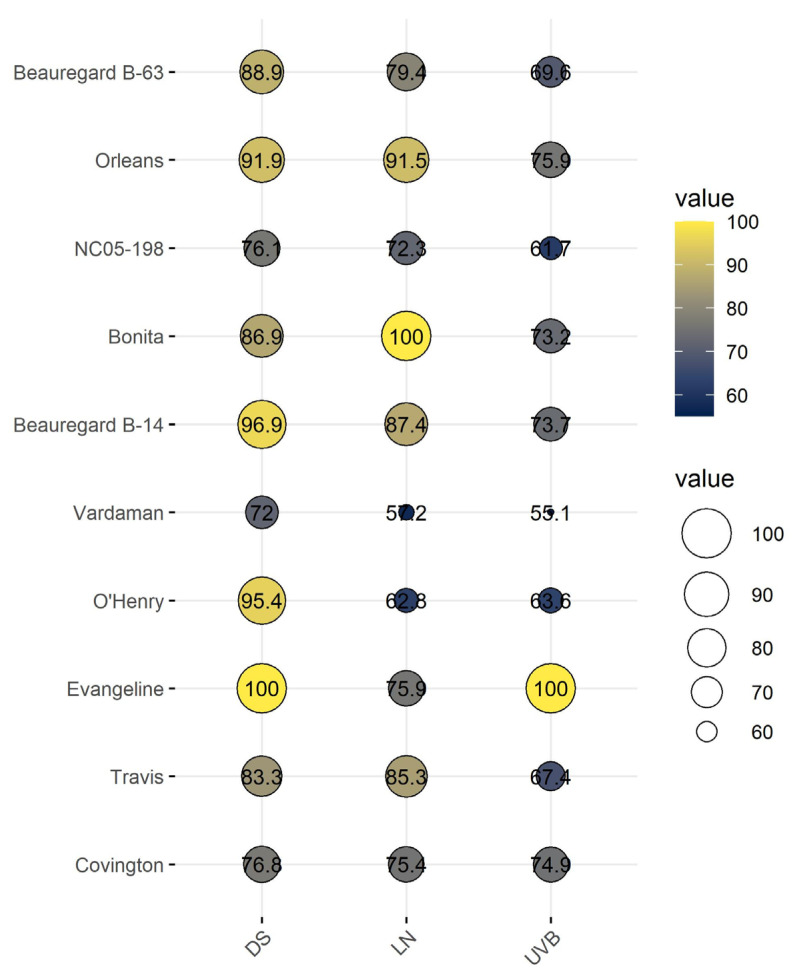
Bubble plot of the cultivars’ total stress indices, calculated from shoot, root, physiological, and gas-exchange traits, of 10 sweetpotato cultivars measured at 20 days after planting under drought (DS), ultraviolet-B (UV-B), and low-nitrogen (LN) stress conditions.

To account for the total early-season vigor of the cultivars, the cumulative stress response index for all ten sweetpotato cultivars was calculated and compared to the relative performance of the cultivars across treatments (Figure 8), as shown in other experiments using rice [43], soybean [79], and corn [80]. The cultivars were classified into three major groups (tolerant, intermediate, and sensitive) based on their stress response indices percentage (Figure 8). The size of the bubbles designated to each cultivar signifies the level of tolerance and total vigor under the given stress treatment. The cultivars such as ‘Evangeline’ and ‘Beauregard B-14’ performed well under DS; ‘Evangeline’ and ‘Orleans’ under UV-B; and ‘Bonita’ and ‘Orleans’ under LN stress. In general, the cultivars ‘Vardaman’ and ‘NC05-198’ performed consistently poorly across stress treatments (Figure 8). The difference between the cultivars indicates the presence of a sizeable genetic diversity in stress adaptive mechanisms.

## 4. Conclusions

The ten sweetpotato cultivars examined in this study exhibited substantial variation in their shoot, root, physiological, and gas-exchange traits under DS, UV-B, and LN stress treatments. All three stress factors caused changes in root and shoot developmental and physiological characteristics in sweetpotato cultivars. The changes that occurred in the traits were not the same across treatments. DS reduced most shoot developmental traits to maintain root growth. On average, DS reduced the shoot traits by 29%, the root traits by 24%, and the physiological and gas-exchange traits by 10%. UV-B stress induced shoot growth and plant pigments and decreased the photosynthetic rate, and the overall change rates were about +1% in the shoot, +4% in the root, +17% in the plant pigment, and −16% in the gas-exchange traits. LN stress induced a moderate decrease in shoot growth (11%) and a markable increase in root growth (18%), and the observations for the other traits were quite similar to those under UV-B stress. Genetic variation and the cultivars’ mean performance of various traits and root yield imply that the more significant leaf number and stem biomass under UV-B and nitrogen stress and the shorter vine length and lower leaf number under DS were ideal plant responses to produce a better yield. Stress-specific tolerant and sensitive cultivars were identified based on the total stress response indices. ‘Evangeline’, ‘O’Henry’, ‘Beauregard B-14’, and ‘Orleans’ under DS; ‘Evangeline’, ‘Covington’, and ‘Orleans’ under UV-B; and ‘Bonita’, ‘Beauregard B-14’, and ‘Orleans’ under LN stress were found to be tolerant. The cultivar ‘Vardaman’ was found to be sensitive across stress treatments. The highly stable and productive cultivars under all four treatments were identified in this study using MTSI and WAASB analyses. This general information could help determine which shoot or root traits are well-suited among the studied sweetpotato cultivars and stress treatments. The cultivars identified as tolerant, sensitive, and well-adapted within and across stress treatments can be used as sources for abiotic stress tolerance breeding programs. However, these cultivar performances must be evaluated under field conditions to validate the outcomes. Phenotyping different genetic resources and mapping genetic loci associated with combined stress responses would help develop climate-ready sweetpotato cultivars for current and future climatic conditions.

## Figures and Tables

**Table 1:** Analysis of variance of the cultivar (C), treatment (T), and their interactions (C × T) for physiological, and shoot- and root-related traits of 10 cultivars of sweetpotato measured at 20 days after transplanting.

Trait	Unit	Drought			Low Nitrogen			Ultraviolet-B		
		C	T_a_	C × T_a_	C	T_b_	C × T_b_	C	T_c_	C × T_c_
Chlorophyll	µg cm^−2^	ns	***	ns	ns	ns	ns	*	***	*
Flavonoids	unitless	ns	ns	**	ns	***	***	*	***	*
Anthocyanin	unitless	***	ns	ns	**	ns	ns	***	***	*
NBI	unitless	ns	ns	**	ns	***	***	**	**	**
Photosynthesis	µmol m^−2^ s^−1^	**	***	ns	ns	***	ns	ns	***	ns
Stomatal conductance	mol m^−2^ s^−1^	ns	***	ns	ns	**	ns	**	**	**
Transpiration	H_2_O m^−2^ s^−1^	ns	***	**	ns	ns	ns	*	**	*
ETR	µmol m^−2^ s^−1^	**	***	ns	*	***	ns	ns	***	*
Ci/Ca		ns	**	ns	ns	**	ns	ns	ns	ns
Minimal fluorescence		ns	ns	ns	ns	***	ns	ns	**	ns
Maximal fluorescence		ns	***	ns	ns	***	ns	ns	***	ns
Steady-state fluorescence		ns	ns	ns	*	***	**	ns	***	ns
Quantum efficiency		*	*	ns	*	ns	ns	ns	***	ns
Vine length	cm	***	*	***	***	ns	**	***	ns	***
Leaf number	no./plant	ns	***	***	ns	**	***	**	*	**
Leaf area	cm^2^	***	***	*	***	**	ns	***	ns	*
Leaf dry weight	g/plant	***	**	**	*	ns	ns	***	ns	**
Total root length	cm	**	***	ns	ns	***	ns	ns	ns	ns
Root surface area	cm^2^	*	***	ns	***	**	ns	ns	ns	ns
Root diameter	mm	***	ns	ns	***	ns	**	***	**	ns
Root volume	cm^3^	**	***	ns	***	**	*	*	ns	ns
Root tips	no./plant	ns	ns	ns	*	***	ns	ns	ns	ns
Root forks	no./plant	**	***	ns	**	*	ns	**	ns	ns
Root crossings	no./plant	***	**	ns	**	*	ns	***	ns	ns
Stem dry weight	g/plant	***	***	**	***	**	ns	***	ns	*
Root dry weight	g/plant	***	ns	ns	***	**	*	**	ns	ns
Root to shoot ratio	ratio	***	***	ns	**	***	*	***	*	ns
Storage root number	no./plant	**	ns	**	***	ns	*	***	ns	ns

T_a_ = drought and control treatment; T_b_ = low-nitrogen and control treatment; T_c_ = ultraviolet and control treatment. *, **, and *** indicate significance levels at *p* < 0.05, *p* < 0.01, *p* < 0.001*,* respectively. ‘ns’ indicates nonsignificant.

## Data Availability

Not applicable.

## Supplementary Materials

The following supporting information can be downloaded at: https://www.mdpi.com/article/10.3390/genes13010156/s1, Table S1: Analysis of variance means of physiological and gas-exchange traits of 10 sweetpotato cultivars measured at 20 days after planting under control, drought (DS), ultraviolet-B (UV-B) radiation, and low-nitrogen (LN) stress conditions, Table S2: Analysis of variance means of shoot and root developmental and biomass-related traits of 10 sweetpotato cultivars measured at 20 days after planting under control, drought (DS), ultraviolet-B (UV-B) radiation, and low-nitrogen (LN) stress conditions.
