# Supplementary material for: Drought, Low Nitrogen Stress, and Ultraviolet-B Radiation Effects on Growth, Development, and Physiology of Sweetpotato Cultivars during Early Season
Source: Genes (Basel). 2022 Jan 16;13(1):156. doi: 10.3390/genes13010156 (PMC8775168; doi:10.3390/genes13010156)
Supplement: Supplementary file 1 [file genes-13-00156-s001.zip › genes-1526846-supplementary.pdf]

Supplementary Material

Table S1. Analysis of variance means of physiological and gas-exchange traits of 10 sweetpotato cultivars measured at 20 days after planting under control, drought (DS), ultraviolet-B (UV-B) radiation, and low-nitrogen (LN) stress conditions.

Trait	Unit	Treatment	Beauregard B-63	Orleans	NC05198	Bonita	Beauregard B-14	Vardaman	O'Henry	Evangeline	Travis	Covington
Chlorophyll	μg cm^{-2}	Control	19.4	22.6	21.5	18.0	22.2	22.2	23.0	23.7	24.0	18.5
		DS	24.7	27.7	25.4	28.9	25.4	23.7	29.0	26.1	24.9	28.3
		LN	23.0	23.5	21.8	20.2	22.7	23.3	21.6	22.1	24.8	21.8
		UV-B	34.4	34.8	23.9	28.2	31.6	23.3	28.7	27.7	27.2	30.0
Flavonoids		Control	0.90	0.46	0.53	0.78	0.61	0.68	0.74	0.80	0.82	0.76
		DS	0.95	0.91	0.83	0.76	0.89	0.67	0.67	0.74	0.64	0.76
		LN	0.97	1.31	0.88	0.83	1.03	0.94	0.98	0.88	0.89	0.84
		UV-B	1.11	1.31	0.98	1.01	1.09	1.09	1.44	0.78	1.29	1.26
Anthocyanin		Control	0.16	0.11	0.11	0.16	0.11	0.11	0.10	0.41	0.14	0.17
		DS	0.13	0.09	0.11	0.11	0.12	0.15	0.09	0.33	0.11	0.14
		LN	0.17	0.17	0.14	0.23	0.18	0.13	0.14	0.22	0.12	0.21
		UV-B	0.06	0.06	0.12	0.08	0.07	0.12	0.09	0.13	0.10	0.09
NBI		Control	24.0	57.2	48.0	23.5	39.7	32.9	31.7	32.0	31.2	24.8
		DS	28.0	30.5	30.6	39.7	28.4	35.4	46.4	37.6	45.4	39.0
		LN	24.6	18.2	25.1	24.6	22.2	25.3	23.2	26.1	28.6	27.3
		UV-B	31.5	27.2	28.1	31.5	29.8	21.8	20.7	39.3	21.4	24.0
Photosynthesis	μ mol m^{-2} s^{-1}	Control	32.2	29.6	31.8	32.7	34.0	33.1	31.9	31.1	34.9	30.7
		DS	23.5	22.2	22.7	11.9	24.1	25.4	26.6	22.6	23.8	11.1
		LN	28.5	33.2	26.6	31.2	27.6	29.6	25.4	28.5	30.6	26.7
		UV-B	23.2	22.7	32.9	22.9	26.8	19.7	24.3	28.0	17.4	28.4
Stomatal conductance	mol m^{-2} s^{-1}	Control	0.55	0.49	0.52	0.64	0.61	0.53	0.60	0.54	0.52	0.69
		DS	0.39	0.34	0.35	0.21	0.37	0.47	0.37	0.31	0.31	0.21
		LN	0.55	0.58	0.36	0.52	0.49	0.39	0.39	0.41	0.40	0.47
		UV-B	0.43	0.40	0.70	0.37	0.49	0.30	0.43	0.78	0.30	0.51
Transpiration	H_2O m^{-2} s^{-1}	Control	7.63	7.38	8.22	9.50	8.30	8.24	9.34	7.80	8.07	8.96
		DS	7.22	6.56	6.41	4.62	6.99	7.72	6.92	6.49	6.40	4.67
		LN	9.56	10.12	7.65	9.40	8.68	7.80	7.75	8.29	8.11	8.96
		UV-B	7.11	6.58	9.60	6.67	7.62	5.61	7.33	9.90	5.40	8.05
ETR	μ mol m^{-2} s^{-1}	Control	233	207	205	211	224	230	218	205	259	208
		DS	189	198	175	120	190	180	223	162	200	110
		LN	182	219	190	208	192	209	160	183	214	195
		UV-B	199	193	214	178	222	170	191	203	140	204
Ci/Ca		Control	0.74	0.71	0.72	0.77	0.75	0.72	0.76	0.74	0.70	0.79
		DS	0.73	0.70	0.69	0.75	0.71	0.74	0.68	0.68	0.66	0.71
		LN	0.76	0.74	0.67	0.72	0.71	0.66	0.69	0.69	0.66	0.74
		UV-B	0.76	0.74	0.78	0.70	0.75	0.71	0.75	0.82	0.73	0.75
Minimal fluorescence		Control	76.1	84.1	77.0	73.4	73.9	77.1	73.8	76.1	78.3	78.6
		DS	67.7	75.1	70.8	68.7	74.7	48.9	75.7	76.8	75.5	74.7
		LN	538	539	552	547	532	566	598	580	554	534
		UV-B	64.6	61.5	70.3	76.7	70.6	60.6	71.4	75.6	74.3	76.5
Maximal fluorescence		Control	174	189	171	153	166	181	161	161	197	177
		DS	138	148	142	121	150	173	166	150	160	132
		LN	1086	1222	1166	1178	1126	1320	1444	1263	1287	1116
		UV-B	133	125	155	145	150	132	139	158	139	162
Steady-state fluorescence		Control	110	126	115	101	107	116	106	110	117	119

	DS	94	102	103	98	105	124	108	111	109	110
	LN	785	811	827	804	796	896	1092	913	866	781
	UV-B	89.6	85.8	103.0	103.7	97.8	97.7	96.5	108.2	108.1	109.5
Quantum efficiency	Control	0.56	0.55	0.55	0.51	0.55	0.57	0.54	0.53	0.60	0.56
	DS	0.49	0.49	0.49	0.43	0.50	0.72	0.54	0.48	0.52	0.42
	LN	0.50	0.56	0.52	0.53	0.53	0.57	0.58	0.54	0.57	0.52
	UV-B	0.50	0.49	0.55	0.47	0.52	0.54	0.48	0.52	0.46	0.52

Table S2. Analysis of variance means of shoot and root developmental and biomass-related traits of 10 sweetpotato cultivars measured at 20 days after planting under control, drought (DS), ultraviolet-B (UV-B) radiation, and low-nitrogen (LN) stress conditions.

**Trait**	**Unit**	**Treatment**	**Beauregard B-63**	**Orleans**	**NC05198**	**Bonita**	**Beauregard B-14**	**Vardaman**	**O'Henry**	**Evangeline**	**Travis**	**Covington**
Vine length	cm	Control	39.3	39.9	20.9	55.8	27.9	36.1	60.0	32.0	34.5	16.6
		DS	36.5	26.3	11.6	22.6	40.9	16.5	41.0	42.5	19.8	18.0
		LN	51.3	30.1	12.9	35.4	57.3	22.5	32.6	21.8	23.9	26.6
		UV-B	45.5	33.9	11.1	38.3	47.0	21.8	59.1	66.0	33.5	32.3
Leaf number	no./plant	Control	10.00	9.00	9.25	11.00	7.25	10.75	11.00	9.00	8.25	8.25
		DS	8.25	8.25	6.50	6.75	8.25	6.25	8.00	9.25	6.75	6.50
		LN	9.50	9.25	7.50	8.75	10.25	7.00	7.25	8.00	7.75	8.00
		UV-B	10.50	10.50	8.00	10.25	9.50	8.50	11.25	13.25	10.00	9.25
Leaf area	cm^2	Control	520	747	928	1055	530	799	672	557	704	586
		DS	407	504	546	621	389	417	506	505	571	278
		LN	550	670	687	929	494	556	412	410	818	473
		UV-B	472	754	741	805	453	535	536	749	872	715
Leaf dry weight	g/plant	Control	2.22	2.50	3.63	4.05	1.89	3.19	2.66	1.76	2.65	2.47
		DS	1.64	1.72	1.95	2.10	1.36	1.43	1.81	1.66	1.86	1.27
		LN	2.24	3.22	3.08	4.13	2.12	2.38	1.70	1.72	3.64	2.26
		UV-B	2.03	3.17	2.99	3.12	1.78	2.20	2.53	3.04	3.43	2.80
Total root length	cm	Control	5188	5581	5513	4537	4560	4820	5250	3871	4463	5463
		DS	3695	4364	3450	3483	3612	2540	4448	3158	3170	2748
		LN	5949	5506	6614	6796	6061	4053	5462	5097	6370	5848
		UV-B	5301	5501	5073	5117	4740	3626	5067	5601	3698	5469
Root surface area	cm^2	Control	825	870	927	957	653	883	871	584	790	1006
		DS	582	753	543	675	551	437	718	454	528	498
		LN	1023	1242	1036	1497	919	680	776	689	1236	970
		UV-B	746	889	752	821	655	562	740	923	633	950
Root diameter	mm	Control	0.50	0.50	0.54	0.67	0.45	0.58	0.53	0.47	0.56	0.58
		DS	0.50	0.55	0.50	0.61	0.49	0.54	0.51	0.46	0.54	0.57
		LN	0.54	0.73	0.49	0.70	0.48	0.52	0.45	0.43	0.61	0.53
		UV-B	0.45	0.51	0.47	0.51	0.44	0.49	0.47	0.51	0.55	0.54
Root volume	cm^3	Control	10.55	10.81	12.45	16.87	7.47	12.96	11.51	7.05	11.16	15.16
		DS	7.31	10.40	6.83	10.48	6.75	6.02	9.30	5.20	7.02	7.25
		LN	14.3	22.6	13.2	26.7	11.3	9.09	8.88	7.41	19.4	12.9
		UV-B	8.37	11.5	8.90	10.5	7.23	6.93	8.64	12.2	8.66	13.2
Root tips	no./plant	Control	6774	7432	7480	6233	7313	6637	5725	4403	7143	6186
		DS	5638	6054	5149	5228	7113	4367	7031	4858	5599	5153
		LN	8911	8379	13474	11182	14103	5983	10863	9180	10233	9885
		UV-B	7865	8176	7659	8721	7172	7664	6353	6003	5715	6274

Root forks	no./plant	Control	46873	54669	54022	44726	35413	38463	56885	28866	36028	48995
		DS	24642	28514	18764	19280	23725	12049	28971	18868	17942	15680
		LN	56066	59179	61925	76503	53353	27730	46459	39565	56793	46192
		UV-B	40751	57298	44278	42770	32854	21656	41032	48683	22029	41917
Root crossings	no./plant	Control	4238	4994	4515	2547	3532	2792	4682	2693	2782	3752
		DS	2226	2329	1859	1453	2106	1080	2490	1944	1566	1187
		LN	4303	3474	5658	4335	4701	2326	4301	4058	3835	3669
		UV-B	4307	4740	4343	3716	3424	2138	4076	3836	1934	3663
Stem dry weight	g/plant	Control	1.88	2.23	2.31	3.64	1.48	2.02	2.69	1.24	2.09	1.78
		DS	1.72	1.75	1.40	1.84	1.35	1.02	2.17	1.44	1.69	1.18
		LN	1.93	2.45	1.77	2.84	1.57	1.26	1.49	0.95	2.11	1.39
		UV-B	1.67	2.52	1.78	2.61	1.63	1.37	2.20	2.26	2.41	2.26
Root dry weight	g/plant	Control	1.27	1.10	1.49	1.64	0.80	1.19	1.25	0.63	1.09	1.58
		DS	1.24	1.62	1.28	1.61	0.98	0.62	1.41	0.82	1.04	0.98
		LN	1.52	2.67	1.78	2.82	1.21	0.85	0.92	0.78	1.72	1.29
		UV-B	0.89	1.46	1.14	1.20	0.70	0.68	1.11	1.21	0.93	1.36
Root to shoot ratio		Control	0.31	0.23	0.25	0.21	0.24	0.23	0.23	0.21	0.23	0.37
		DS	0.37	0.47	0.38	0.41	0.36	0.25	0.36	0.26	0.29	0.40
		LN	0.36	0.47	0.37	0.41	0.33	0.23	0.29	0.29	0.30	0.35
		UV-B	0.24	0.26	0.24	0.21	0.21	0.19	0.23	0.23	0.16	0.27
Storage root number	no./plant	Control	9.75	9.25	7.75	3.75	10.0	4.75	3.00	8.33	2.75	12.25
		DS	4.75	6.25	6.50	6.25	4.00	5.00	6.50	5.75	5.00	8.50
		LN	7.50	12.25	8.50	9.00	6.50	4.00	5.00	6.00	9.25	9.50
		UV-B	9.00	11.00	7.25	5.50	7.25	3.50	3.25	10.75	4.25	11.75
